# A pre–post study of self-esteem, self-concept and body image among adolescents in the context of a co-created community intervention on screen use

**DOI:** 10.1371/journal.pone.0360373

**Published:** 2026-10-15

**Authors:** Eva Artigues-Barberà, Mercè Pollina-Pocallet, Ester García-Martínez, Joaquim Sol, Aleix Ayats Pallés, Marta Ortega Bravo

**Affiliations:** 1 Department of Nursing and Physiotherapy, Faculty of Nursing and Physiotherapy, University of Lleida, Lleida, Spain; 2 Atenció Primària, Gerència Territorial de Lleida, Institut Català de la Salut, Lleida, Spain; 3 Fundació Institut Universitari per a la Recerca a l’Atenció Primària de Salut Jordi Gol i Gurina (IDIAPJGol), Barcelona, Spain; 4 Centre d’Atenció Primària Bellpuig, Institut Català de la Salut, Bellpuig, Lleida, Spain; 5 Health Care Research Group (GRECS), Biomedical Research Institute of Lleida (IRBLleida), Lleida, Spain; 6 Metabolic Physiopathology Research Group, Department of Experimental Medicine, Biomedical Research Institute of Lleida (IRBLleida), Lleida, Spain; 7 Centre d'Atenció Primària Almacelles, Institut Català de la Salut, Almacelles, Lleida, Spain; 8 Department of Medicine, Faculty of Medicine, University of Lleida, Lleida, Spain; West University of Timisoara: Universitatea de Vest din Timisoara, ROMANIA

## Abstract

**Introduction:**

Excessive screen use negatively affects adolescents’ self-esteem, self-concept, and body image, largely due to social comparison on digital platforms. Educational interventions are often less effective without community involvement, highlighting the importance of an ecological and participatory approach. This study aimed to assess the changes observed following the implementation of a community co-created intervention programme on screen use, self-esteem, self-concept, and positive body image among adolescents from a rural area in Catalonia.

**Methods:**

The study was conducted in Bellpuig, a rural municipality in Catalonia, Spain, involving adolescents aged twelve to sixteen years enrolled at `Lo Pla d’Urgell´ institute. This was a quasi-experimental single-group study with pre and post intervention measurements. The intervention program was co-created with adolescents, families, teachers, healthcare professionals, and local community stakeholders through a participatory design process, and comprised educational and participatory activities focused on fostering digital balance and promoting self-esteem, self-concept, and positive body image. Data were collected using validated self-report measures before and after the intervention. Pre–post changes were assessed using two-sided Wilcoxon signed-rank tests. Differences in individual pre–post change scores between girls and boys were assessed using two-sided Mann–Whitney U tests. Rank-biserial correlations with 95% confidence intervals were reported as effect-size estimates.

**Results:**

The intervention led to a moderate improvement in positive body image, self-esteem, and self-concept among adolescents. No notable differences were found in the impact of the intervention between sex participants. However, baseline differences in self-esteem and body image were observed, with higher initial values among boys.

**Conclusion:**

These findings suggest that a community co-created intervention program can be an effective strategy to improvement positive body image and self-esteem among adolescents. Future research should explore long-term effects and adaptation to diverse populations.

**Public Contribution:**

This study underscores the value of community co-created interventions to support adolescent well-being, especially in rural areas with limited mental health resources. The findings offer useful guidance for health professionals, educators, and policymakers to design and evaluate programs that foster healthy self-perception and digital habits, while also raising community awareness.

## Introduction

Adolescence is a critical stage in human development, characterized by a complex interaction of biological, psychological, and social factors [1]. During this period, self-concept, self-esteem, and body image play a fundamental role in the construction of adolescents’ personal and social identity [2,3]. Although these concepts are interconnected and complement each other, they are not equivalent [3].

Self-concept refers to the perception and image a person has of themselves from a cognitive standpoint, while self-esteem encompasses the affective evaluation of that self-image, or how a person feels about themselves based on that perception [3]. In this process, body image—the mental representation each individual constructs about their own body—directly influences both self-concept and self-esteem [4,5]. A positive body image is not simply the absence of body dissatisfaction [5]; it involves acceptance, respect, and appreciation for one's body, regardless of its actual physical appearance, constituting a protective factor for mental and physical Health [6,7].

The use of screens and digital devices (mobile phones, televisions, gaming consoles, computers, etc.) has become a central aspect of adolescents’ lives, significantly influencing their psychological, emotional, and social development [8]. While technology offers benefits such as learning and socialization [9], excessive use negatively impacts self-esteem, self-concept, and body image, primarily due to social comparison and exposure to unattainable beauty ideals on digital platforms [10]. This phenomenon can lead to dissatisfaction with one's body image and affect the construction of personal identity, compromising psychosocial well-being [10]. In recent years, screen use and digital devices have increased considerably among adolescents. According to 2023 data from the National Observatory of Technology and Society, 70.6% of children aged 10–15 in Spain have a mobile phone [11] and spend between 4 and 6 hours a day using it [12]. Among the most common activities performed on these devices is social media use [13]. Several studies have shown associations between social media use by adolescents and increased concerns about body image [14], as well as higher levels of body dissatisfaction, especially among girls compared to boys [15,16].

Adolescents frequently encounter highly edited images of peers, celebrities, and influencers, which promotes the internalization of unrealistic beauty standards and fosters social comparison [17]. This phenomenon can exacerbate body dissatisfaction, negatively affecting self-esteem and self-concept [18]. As a result, this may manifest in increased negative mood, anxiety, and depression, and even in eating disorders [17] and sleep disorders [19]. Moreover, it can negatively affect adolescents’ academic performance and hinder their ability to form healthy relationships [13,20]. In this regard, high levels of screen time, especially due to internet and social media use [21–23], have been associated with mental health problems in adolescents. This underscores the urgent need to implement educational programs that help both adolescents and their families manage and balance screen use in a healthy way [24], promoting high self-esteem, a healthy self-concept, and a positive body image.

Bronfenbrenner's ecological model [25] offers a theoretical framework that views individual behavior as the product of complex interactions and influence relationships across different levels: interpersonal, organizational, community, and public policies. This approach emphasizes that the way adolescents perceive themselves and their own bodies is not an isolated phenomenon, but is deeply conditioned by sociocultural factors that act within their immediate and broader environment [26,27]. In rural communities, interactions between people tend to be more intimate and close due to geographic proximity. However, this closeness can also entail stricter social norms, creating greater pressure to conform to certain beauty ideals. Moreover, in these communities, where access to educational and mental health resources is more limited, adolescents may be more exposed to these pressures, intensifying the negative effects on their self-esteem and self-concept. This combination of social closeness and scarce professional support can create a challenging environment for adolescents, who struggle to balance social expectations with their own identity. In this context, emotional support from family and positive interactions with peers and educators are essential for building a healthy self-concept and mitigating the negative effects of social pressures [26]. Thus, adolescents’ self-esteem, self-concept, and body image are strongly influenced by the context in which they develop, family dynamics, interactions with friends, school experiences, and, crucially, the media representations they encounter on social media and other digital platforms.

Despite growing concerns about the adverse effects of problematic screen use, to the best of our knowledge, many interventions aimed at adolescents to improve self-esteem, self-concept, and positive body image have followed an individualistic approach, primarily implemented in the school setting through preventive or therapeutic programs [28–30]. However, evidence suggests that, to achieve significant and lasting change, strategies are needed that involve not only adolescents but also their families, educators, and the community at large [26]. In this context, co-creating interventions with the community has emerged as a promising approach, tailoring strategies to meet the needs of adolescents in rural areas and encouraging their engagement [31,32]. Co-creation is a participatory approach to creative and interactive problem-solving among various stakeholders with a shared goal and a shared decision-making process, from the collaborative identification of problems and the generation of solutions to implementation and evaluation [33]. Therefore, co-created interventions involving multiple agents may be more effective in achieving high self-esteem, a healthy self-concept, and a positive body image in adolescents.

In response to these challenges, the present study proposes a participatory research approach centered on the ecological model, aiming to develop an intervention program that promotes healthy screen use, thereby enhancing adolescents’ psychological well-being. It is expected that the implementation of this program will not only contribute to improving self-esteem, self-concept, and positive body image but will also provide evidence on the effectiveness of co-created interventions in promoting digital and psychosocial well-being. This approach will allow for the development of inclusive and sustainable strategies in this field, ensuring that initiatives are effective and responsive to the real needs of adolescents living in rural areas and their communities [31,32].

This study aimed to assess the changes observed following the implementation of a community co-created intervention programme on screen use, self-esteem, self-concept, and positive body image among adolescents from a rural area in Catalonia. Through participatory research [31,32], the study seeks to actively involve adolescents, their families, and educational staff in designing and implementing the program, ensuring its relevance and effectiveness in its ecological context.

## Methods

### Design

Quasi-experimental single-group study with pre- and post-intervention measurements. This design was considered appropriate given the community-based nature of the intervention.

Since the study aimed to include all adolescents, their families, their educators, and the community as a whole, without excluding any citizens, it was not possible to establish a control group. Logistical limitations also played a significant role, as the study was conducted in a municipality with only one secondary school. Furthermore, some of the healthcare professionals involved occasionally carried out group interventions for health promotion, health education, and disease prevention during adolescence as part of the public health system's service portfolio, which could have influenced the studied variables. These professionals were shared among different healthcare centres in the rural area, which had characteristics similar to those of the intervention municipality. Restricting access to community activities for participants who would belong to a control group was not feasible.

This study adhered to the Transparent Reporting of Evaluations with Nonrandomised Designs (TREND) guidelines [34].

### Setting and study population

The study was conducted in the municipality of Bellpuig, a rural area in the province of Lleida, located in the comarca of Urgell, Catalonia (Spain). In 2023, Bellpuig had a population of 5,182 inhabitants within an area of 34.98 km², of whom 2,748 were male and the remainder female. In 2024, Bellpuig had a population of 297 inhabitants aged 12–16 years, of whom 144 were male and 153 females [35].

The study population consisted of adolescents enrolled at `Lo Pla d’Urgell´ institute during the 2023/2024 academic year. Students from the first to the fourth year of Compulsory Secondary Education (CSE), aged between 12 and 16 years of both sexes, were included. Adolescents whose parents or legal guardians did not provide informed consent for participation were excluded from the study.

### Sampling and participant recruitment

The recruitment period lasted from 06-11-2023 to 30-11-2023. Non-probability convenience sampling was employed, selecting adolescents who met the established eligibility criteria for the research. The school nurse sought permission from the school administration to conduct the study.

Communication with families was carried out exclusively through the channels established by the school, as the school nurse was not authorised by the school administration to contact them directly to inform them about the study. The school administration sent an email to families on behalf of the research team, detailing the study's objectives and inviting them to participate, attaching the study information sheet and informed consent form. Parents or legal representatives who authorised the adolescents’ participation signed the informed consent form and submitted it to the school administration, which stored it securely until it was handed over to the school nurse, who was part of the research team.

### Development of the community interventions programme

To develop the community intervention programme, the conceptual framework of the Analysis, Design, Development, Implementation, and Evaluation (ADDIE) model was applied [36].

In the **analysis phase**, the community health assessment conducted by the local health centre identified three main health priorities in Bellpuig: loneliness, immigration, and adolescent mental health. Among these, adolescent mental health emerged as a growing concern within the community. The research team reviewed the existing scientific literature on the variables studied in adolescence, along with the results of previous studies in the area, in order to interpret and understand the relevant information for the research and to identify the health needs and problems detected in the local context of the studied population.

In the **design phase**, the school nurse contacted the child and adolescent health table, composed of 25 representatives from the town council, the comarca council, the administrations of schools and the secondary school, the psychopedagogical services of the educational centres, parent associations, families, school nurses, and other healthcare professionals from the health centre, as well as sports and leisure entities, and the adolescents themselves from the municipality of Bellpuig, to invite them to participate in the design and development of an intervention programme aimed at improving self-esteem, positive body image, and self-concept among adolescents. Subsequently, in this phase, a joint assessment of the community's needs and concerns was carried out, and these were prioritised to design specific actions and interventions. After three monthly sessions, the use of screens was prioritised as the central theme for the community intervention programme, and the resources and initiatives that could be contributed by each participant and institution were identified.

In the **development phase**, five participatory design cycles were conducted to integrate evidence-informed activities with the concerns expressed by the child and adolescent health table and the feasible resources identified by the participating stakeholders, reaching consensus among the group. The intervention programme “Child and Adolescent Mental Health: Screens and Digital Balance” was established with the active participation of the child and adolescent health table. This programme was developed during the period from September to December 2023. Following the initial proposals developed in the working meetings, a final protocol was elaborated detailing the type of intervention, the target audience, the objectives, the methodology, the content, the duration, and the material resources. The content was validated by the research team, which consisted of three nurses (one specialised in school health, and another who is also a psychopedagogue) and a community psychologist, based on the available scientific evidence and clinical experience, while considering the specific characteristics and needs of the rural context. Subsequently, the logistical coordination schedule with the collaborating entities was developed, along with the dissemination process that included the creation and distribution of advertising posters.

In the **implementation phase**, the community intervention programme “Child and Adolescent Mental Health: Screens and Digital Balance” was carried out with various activities in the municipality of Bellpuig during the period from December 2023 to May 2024.

Finally, in the **evaluation phase**, the subsequent effect of this programme on self-esteem, self-concept, and positive body image in the adolescent population enrolled at `Lo Pla d’Urgell´ institute in any of the four years of CSE during the 2023/2024 academic year in Bellpuig was assessed.

### Study procedure

#### Pre-intervention phase.

Before commencing the community intervention programme, adolescents whose parents or legal representatives provided informed consent and self-completed the Data Collection Notebook (DCN) in a designated classroom at the school during the first week of December 2023. The school nurse was responsible for distributing the booklet in paper format to the adolescents and assisting them by explaining the various scales and addressing any questions regarding the items.

#### Intervention phase.

The community intervention programme ‘Child and Adolescent Mental Health: Screens and Digital Balance’ was conducted in the municipality of Bellpuig, with a duration of six months. The programme included a total of 6 sessions directed at the general community and three specific sessions for the adolescent population, held in the school environment (`Lo Pla d’Urgell´ institute). Each session addressed a different theme and lasted between 60 and 120 minutes. To facilitate maximum attendance from families, childcare was provided for infants during sessions held on weekdays from 6 p.m. onwards, and extensive promotion was carried out through social media, posters, a local magazine, and announcements from the municipality and the town council.

Table 1 and Table 2 details the objectives, content, description of the various sessions and promotional posters distributed with the collaborating entities.

**Table 1:** Sessions of the Monograph on Screens and Digital Well-being (ICT and social media).

Session Focus	Content	Methodology-Description
**Meeting “Child and adolescent health table”** • Bring together the members of the Child and Adolescent Health Table, composed of representatives from the Parents’ Association, the Town Hall, the Community Action Plan of the Regional Council, Youth of the Regional Council, the Primary Care Centre, as well as the management and the psycho-pedagogical team of the schools in Bellpuig. • Agree on the key topics to address collectively, with the aim of designing and offering specific training for parents.	**1. Evaluation of Actions Taken in 2022/23.** **2. Current Needs and Reality.** Various concerning issues are discussed, including: the need for spaces where parents can share activities with their children; the growing impact on self-esteem and emotional well-being in primary education; the increasing difficulty in overcoming life challenges; the rising delay in language learning caused by the use of ICT; family disorientation and overprotection; concerns regarding sexualised behaviour and pornography consumption; and the challenges posed by social media. **3. Planning of Interventions for This Year.** It is decided to dedicate the Monographic Session to Screens and Digital Well-being (ICT and social media).	**Participatory community meeting**. An open dialogue space is promoted where the invited **entities** can express the vulnerabilities and challenges currently present in the municipality, identifying possible projects to develop based on the needs of the children and adolescents in the area. Led by the members of “Child and adolescent health table”. Held in the municipal conference hall in **September 2023**, with a duration of **2 hours**.
**(1) Connecting with Balance: Let’s Organize and Support Each Other to Regulate Screen Use** • Raise awareness among the population about the importance of starting a community project to prevent the negative consequences of screen use. • Promote the available community resources to manage digital consumption. • Encourage community association through the use of the Telegram app.	**1. What is Community Health?;** 1.1 Phases of Creating a Community Health Project; 1.2 Identification of Key Stakeholders in the Municipality to Work on Child and Youth Emotional Health; 1.3 Community Model as a Driver of Change **2. Definition of Self-Esteem;** 2.1 Bronfenbrenner's Ecological Model and the Development of Children's Identity and Self-Perception; 2.2 Context as a Key Factor in the Development of Self-Esteem **3. Community Associationism;** 3.1 Ways to Promote; Interdepartmental Communication; 3.2 Strategies to Maintain Participation	**Round table** aimed at the **general population**, especially families of adolescents. Led by a member of the “Adolescence Free of Mobile Phones” platform from the Plana de Lleida; the psychologist and the pediatric nurse from the primary healthcare centre; and representatives of the parents’ association of the educational centres of Bellpuig. Held in the municipal conference hall in **December 2023**, with a duration of **1.5 hours**.
**(2) Mobile Phones. Cyber Addiction and the Attention Industry. Where Are We?** • Provide families with resources to manage ICT at home (web applications, apps, etc.). • Raise awareness about how mobile phone use affects the attention of children and adolescents.	**1. Are mobile phones and social networks addictive?** What is the attention industry? **2. Why the misuse of mobile phones** can lead to our children becoming cyber-addicts. **3. How cyber-addiction can affect** the cognitive and physical development of children. **4. Helping parents understand** how different online platforms and social networks compete to capture our attention. **5. Need to learn how to apply prevention and supervision measures** for screen use and to provide effective guidance.	**Conference with discussion** for the resolution of doubts and the expression of concerns. Aimed at the **general population**, especially families of adolescents. Led by a digital trainer and consultant specialising in digital literacy. Held in the municipal conference hall in **January 2024**, with a duration of **1.5 hours**.
**(3) “How to avoid conflicts and headaches with children's screens” Family Coexistence and Screen Use** • Provide guidelines to understand that children's behaviour is the result of their relationship with adults, primarily with their parents. • Present the opportunities for our and their relationship with screens to be valuable sources of learning. Help plan the style of dialogue regarding screens to minimize conflicts.	**1. Factors Affecting Screen Use**; 1.1 Social and Cultural Context; 1.2 Emotional Needs of Children **2. Children's and Adolescents’ Behaviour**; 2.1 How Adults Shape Behaviour; 2.2 Family Dynamics and Their Impact; 2.3 Promotion of Joint Activities **3. Intrafamily Conflicts Regarding Screen Use**; 3.1 Dialogue Style and Handling Techniques; 3.2 Setting Clear and Consistent Rules; 3.3 Emotional Validation Technique **4. Resources of Interest for the General Public**	**Conference with discussion** for the resolution of doubts and the expression of concerns. Aimed at the **general population**, especially families of adolescents. Led by a teacher, pedagogue and assessor of educational and family centres. Held in the municipal conference hall in **February 2024**, with a duration of **1.5 hours**.
**(4) Let's talk about screens** • Reflect on the role of parents in managing conflicts related to screen use. • Provide effective communication strategies and validation.	**1. Emotional bonding in the digital era**; 1.1 Impact of screens on bonding **2. Preparation for mobile phone use**; 2.1 Gradual preparation; 2.2 Dialogue style and management techniques **3. Mobile phone usage agreements**; 3.1 Agreed rules; 3.2 Application of contingencies **4. Resources and strategies for families**	**Workshop with discussion** for the resolution of doubts and the expression of concerns. Aimed at the **general population**, especially families of adolescents. Led by a trainer from the ‘Connecta i Actua’ programme. Held in the municipal conference hall in **March 2024**, with a duration of **1.5 hours**.
**(5) The 21st Century Heroine** • Inform families about the technological reality. • Prevent problems associated with the use of technology. • Provide problem-solving strategies. • Address legal and behavioural questions.	**1. Current context of mobile phone use**; 1.1 Statistics on the increase in usage; 1.2 Common issues arising from usage and overuse; 1.3 Main causes, consequences and signs of addiction risk **2. Guidelines for action**; 2.1 How to assess conflictive situations; 2.2 When to introduce the first devices **3. Resolution of legal issues and behavioural** **4. The adolescent's social role and self-esteem** **5. Community prevention strategies**; 4.1 Prevention and information on the real situation of children in relation to new technologies.	**Conference with real case presentations and debate** to resolve doubts and express concerns. Distribution of a **reinforcement leaflet** with recommendations to follow and avoid. Aimed at the **general population**, especially families of adolescents. Led by an expert psychologist in addiction to new technologies. Held in the municipal conference hall in **April 2024**, with a duration of **1.5 hours**.
**(6) Theatre “OFF”** • Raising awareness among families and children about the harmful use of screens and their impact on interpersonal relationships.	Street theatre play in which two friends meet after many years. They reminisce about their childhood when they used to play on the street, often with a ball. One of the friends brings a gift for the other, who spends the entire play glued to his mobile phone. The ignored friend spends the whole time trying to get his companion's attention, while the addictive power of social networks becomes increasingly evident. In the end, the distracted friend buys a series of mobile phones in search of a better one. Finally, faced with the disinterest of his phone-obsessed friend, the gift-bearing friend leaves alone, leaving the gift behind — the ball they used to play with as children — without the other even noticing.	**Theatre play** aimed at the **general population**, especially families and adolescents. Led by the company ‘Fadunito’. Held on a stage in the municipal central square in **May 2024**, with a duration of **1 hour**.

ICT: Information and Communication Technologies.

**Table 2:** Specific sessions for adolescents at school.

Session Focus	Content	Methodology-Description
**Training Students as “Emotional Delegates”** • Train emotional delegates on the community resources available in the region so they can share this information with their peers.	A meeting is held at the school with the psychopedagogue, inviting emotional delegates—students from each class who have volunteered for the role. They are informed about the resources available in the community to promote emotional well-being and are encouraged to pass this information on to their peers or remind them when necessary.	**Theoretical-practical workshop** aimed at **adolescents** in school. Led by the psychologist from the primary care centre and educationalist of the educational centre. Held at the school in **January 2024**, with a duration of **1 hour**.
**Workshop: Self-Esteem (3rd of CSE)** • To understand what good qualities others think I have.	10 minutes of **theory to focus the topic**: Everyone has good qualities that sometimes go unnoticed. **Activity:** All participants sit in a circle. The first person holds a ball of yarn and throws it to a classmate, saying a good quality about them. The yarn is passed around the circle until everyone has received the yarn and been complimented. A web of yarn will form. Finally, conclusions on individual self-esteem and its impact on the group (self-esteem/bullying) will be drawn.	**Theoretical-practical workshop** aimed at **adolescents** in the 3rd year of CSE^1^, generally between the ages of 14 and 15. Led by the paediatric nurse from the primary care centre. Held at the school in **April 2024**, with a duration of **1 hour**.
**Workshop: Self-Esteem and Body Image (2nd of CSE)** • Recognize the fictional world of social networks.	10 minutes of **theory to focus the topic**: Sometimes the real self and the virtual self don’t align, leading to frustration. **Activity**: Show Instagram photos while students “like” them. Then, compare each photo with the real version. Followed by a debate on the differences between the image presented online and the reality outside of social media.	**Theoretical-practical workshop** aimed at **adolescents** in the 2nd year of CSE^2^, generally between the ages of 13 and 14. Led by the paediatric nurse from the primary care centre. Held at the school in **May 2024**, with a duration of **1 hour**.

^1^3rd year of Compulsory Secondary Education: This is the third of the four academic years that make up the last compulsory stage of free basic education in Spain. In terms of international equivalence, it corresponds to Year 10 (Key Stage 4) in the United Kingdom or 9th Grade (first year of High School) in the United States. ^2^2nd year of Compulsory Secondary Education: This is the second of the four academic years that make up the last compulsory stage of free basic education in Spain. In terms of international equivalence, it corresponds to Year 9 (Key Stage 3) in the United Kingdom or 8th Grade (final year of Middle School or Junior High School) in the United States.

#### Post-test phase.

At the end of the community intervention programme, following the same procedure as in the pre-test phase, the participating adolescents self-completed the DCN during the second week of June 2024. This process aimed to assess changes in the main variables studied.

### Study variables

- **Self-esteem.** Self-esteem will be measured using the Rosenberg Self-Esteem Scale [37], which consists of 10 items rated on a 4-point Likert scale, ranging from 1 (strongly disagree) to 4 (strongly agree). Five of the items are positively worded, while the remaining five are negatively worded. The ones that are positive are items 1–5. For these, “strongly agree” is equal to 4 points, “agree” is equal to 3, “disagree” is equal to 2, and “strongly disagree” is equal to 1. The ones that are negative are items 6–10. For these, the scoring is reversed. “Strongly disagree” is equal to 4 while “strongly agree” is equal to 1. Possible scores range from 10 to 40. Scores below 25 indicate low self-esteem, scores between 26 and 29 indicate medium self-esteem, and scores between 30 and 40 indicate high self-esteem, which is considered normal.
- **Self-concept.** Self-concept will be measured using the Self-Concept Questionnaire Form 5 [38], which has been proven to be valid and reliable. It assesses five dimensions (academic, social, emotional, family, and physical) through 30 items, with 5 response options (1 meaning never and 5 always). Higher scores indicate a stronger self-concept. The total score of the scale ranges from 30 (minimum) to 150 (maximum), with higher scores being associated with a stronger self-concept.
- **Positive body image.** This will be assessed using the Positive Body Image in Adolescents Scale (PBIAS) [39]. The scale explores the factors that reinforce or hinder the development of a positive body image during adolescence. The PBIAS consists of a total of 15 items grouped into four factors: items 1–6 are included in Factor 1 (body self-appreciation), items 7–9 are included in Factor 2 (body other appreciation), items 10–11 are included in Factor 3 (resilience against media body ideals) and items 12–15 are included in Factor 4 (resilience against negative appearance feedback) [40]. The PBIAS demonstrates good internal consistency, high reliability, and validity. Each item is evaluated using a Likert scale with 7 response options (from strongly disagree to strongly agree). The total score of the scale ranges from 15 (minimum) to 105 (maximum), with higher scores being associated with a more positive body image.
- **Other variables.** Sociodemographic variables—such as sex, age, school, academic year, class group, and eating behaviours (anorexia, bulimia, binge eating disorder, and avoidant restrictive food intake disorder)—will be collected using a self-administered questionnaire.

### Data collection

The study variables were measured before and after the community intervention. For data collection, the research team designed a self-administered DCN, which included sociodemographic information as well as scales for self-esteem, self-concept, and positive body image. The school nurse was responsible for distributing the DCN in paper format to the adolescents in a designated classroom within the school. Additionally, she provided the necessary instructions for its proper self-completion, explaining the various scales and items and addressing any questions. A member of the research team was responsible for coding the data, creating the database while minimising potential biases, and storing it in a password-protected institutional environment to ensure the security of the information.

### Data analysis

Preintervention and postintervention scores were compared using two-sided Wilcoxon signed-rank tests for paired observations. To assess whether individual pre–post changes differed by sex, change scores were calculated as the postintervention score minus the preintervention score and compared between girls and boys using two-sided Mann–Whitney U tests. Rank-biserial correlations (r_rb) with 95% confidence intervals were reported as effect-size estimates [41]. Continuous outcome data are presented as median and interquartile range [Q1, Q3].

Analyses were restricted to participants with complete preintervention and postintervention data for the corresponding outcome; consequently, the analytical sample ranged from 103 to 109 participants. Statistical significance was set at a two-sided p < .05. Analyses of the PBIAS subscales were considered exploratory and no adjustment for multiple comparisons was applied; these results were therefore interpreted cautiously. All analyses were performed using R version 4.3.2.

### Ethics statement

Information about the study was provided to all parents or legal representatives of the adolescents, explaining that participation involved the adolescents self-completing DCN before and after the implementation of the intervention programme on screen use in the community. They were informed that participation was voluntary, with no financial compensation, and were given the opportunity to express any doubts or ask questions before granting their informed consent in writing.

Data confidentiality was assured in accordance with Regulation (EU) 2016/679 of the European Parliament and Council of 27 April 2016. This study adhered to the principles of the Declaration of Helsinki and Good Clinical Practice Guidelines. This study was approved by the Clinical Research Ethics Committee of Primary Care Research Institute (IDIAP) Jordi Gol i Gurina Ethical Committee (registration number 20/154-P) in the meeting held on 29-07-2020.

## Results

The eligibility of the 398 adolescents enrolled in CSE at `Lo Pla d’Urgell´ institute (Bellpuig, Lleida) was assessed to participate in the study. In Spain, CSE comprises four academic years. Of these students, 289 did not participate due to a lack of informed consent from their parents or legal guardians, without providing any reason. Ultimately, a total of 109 adolescents were included and completed both the pre- and post-intervention evaluations, being analysed in the study. Participation varied across the four academic years, with n = 15 in 1st year of CSE, n = 42 in 2nd year of CSE, n = 30 in 3rd year of CSE, and n = 22 in 4th year of CSE (Fig 1).

**Fig 1:**
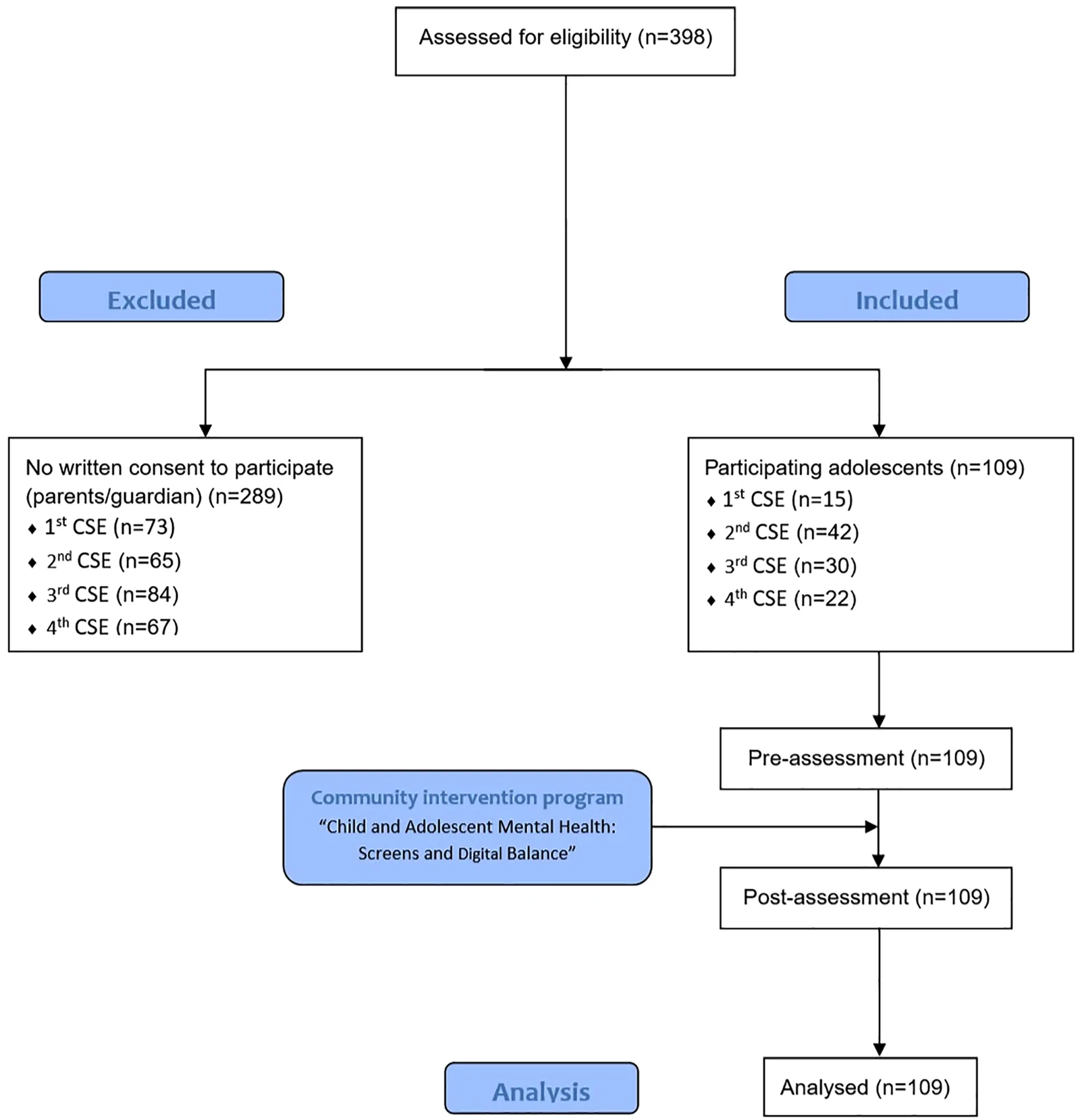
Flow diagram.

### Sample characteristics

Table 3 presents the characteristics of the 109 adolescents who participated in the study, of whom the majority were girls (72.5%, n = 79) and were in 2nd year of CSE (40.5%, n = 32). The mean age of the participants was 13.5 years, with a standard deviation of 1.04. The prevalence of anorexia was 0.9%, bulimia 4.6%, binge eating 11%, and avoidance/restrictions 5.5%. There were no statistically significant differences between sexes for any of the sociodemographic variables.

**Table 3:** Characteristics of the participants (n = 109).

	All sample (n = 109)	Girls (n = 79)	Boys (n = 30)	P value
**Age**	13.5 (1.04)	13.6 (1.03)	13.3 (1.06)	0.327
**Academic year**				0.440
1st of CSE	24 (22.0%)	15 (19.0%)	9 (30.0%)	
2nd of CSE	42 (38.5%)	32 (40.5%)	10 (33.3%)	
3rd of CSE	25 (22.9%)	17 (21.5%)	8 (26.7%)	
4th of CSE	18 (16.5%)	15 (19.0%)	3 (10.0%)	
**Anorexia**	1 (0.92%)	1 (1.27%)	0 (0.00%)	1.000
**Bulimia**	5 (4.59%)	4 (5.06%)	1 (3.33%)	1.000
**Binge eating**	12 (11.0%)	10 (12.7%)	2 (6.67%)	0.505
**Avoidant/restrictive**	6 (5.50%)	6 (7.59%)	0 (0.00%)	0.185

CSE: Compulsory Secondary Education

### Comparison of main variables

Pre–post changes in the main study outcomes were assessed using Wilcoxon signed-rank tests (Table 4, Fig 2). Positive body image increased from a median PBIAS score of 81.00 [70.50, 92.50] at preintervention to 84.00 [75.50, 93.00] at postintervention. The paired analysis indicated a statistically significant pre–post difference (V = 3104.5, p = .028), with a small-to-moderate rank-biserial effect size (r_rb = .254, 95% CI [.038,  .448]).

**Table 4:** Pre–post changes in positive body image, self-esteem, and self-concept, and differences in change by sex.

Outcome	*n*	Pre, Md [Q1, Q3]	Post, Md [Q1, Q3]	Change, Md [Q1, Q3]	*V*	*p*	*r*rb [95% CI]	Girls change, Md [Q1, Q3]	Boys change, Md [Q1, Q3]	*U*	*p*	*r*rb [95% CI]
Positive body image (PBIAS)	103	81.00 [70.50, 92.50]	84.00 [75.50, 93.00]	3.00 [−5.00, 10.00]	3104.5	0.028	0.254 [0.038, 0.448]	4.00 [−4.00, 9.00]	1.00 [−6.75, 12.50]	998.0	0.985	0.003 [−0.250, .255]
Self-esteem	109	29.00 [25.00, 34.00]	31.00 [27.00, 35.00]	2.00 [−1.00, 4.00]	3543.5	< 0.001	0.432 [0.241, 0.590]	1.00 [−1.00, 3.50]	2.00 [−0.75, 4.00]	1088.0	0.511	−0.082 [−0.315, 0.160]
Self-concept	109	106.00 [98.00, 111.00]	106.00 [96.00, 113.00]	2.00 [−4.00, 7.00]	3396.5	0.050	0.221 [0.008, 0.414]	2.00 [−4.00, 6.00]	2.00 [−1.00, 9.00]	1100.5	0.568	−0.071 [−0.305, 0.171]

Values are presented as median [first quartile, third quartile]. Change was calculated for each participant as postintervention minus preintervention. Overall pre–post changes were assessed using two-sided Wilcoxon signed-rank tests. Differences in individual change scores between girls and boys were assessed using two-sided Mann–Whitney U tests. rrb = rank-biserial correlation; CI = confidence interval; PBIAS = Positive Body Image in Adolescents Scale. Analyses were restricted to participants with complete preintervention and postintervention observations for the corresponding outcome.

**Fig 2:**
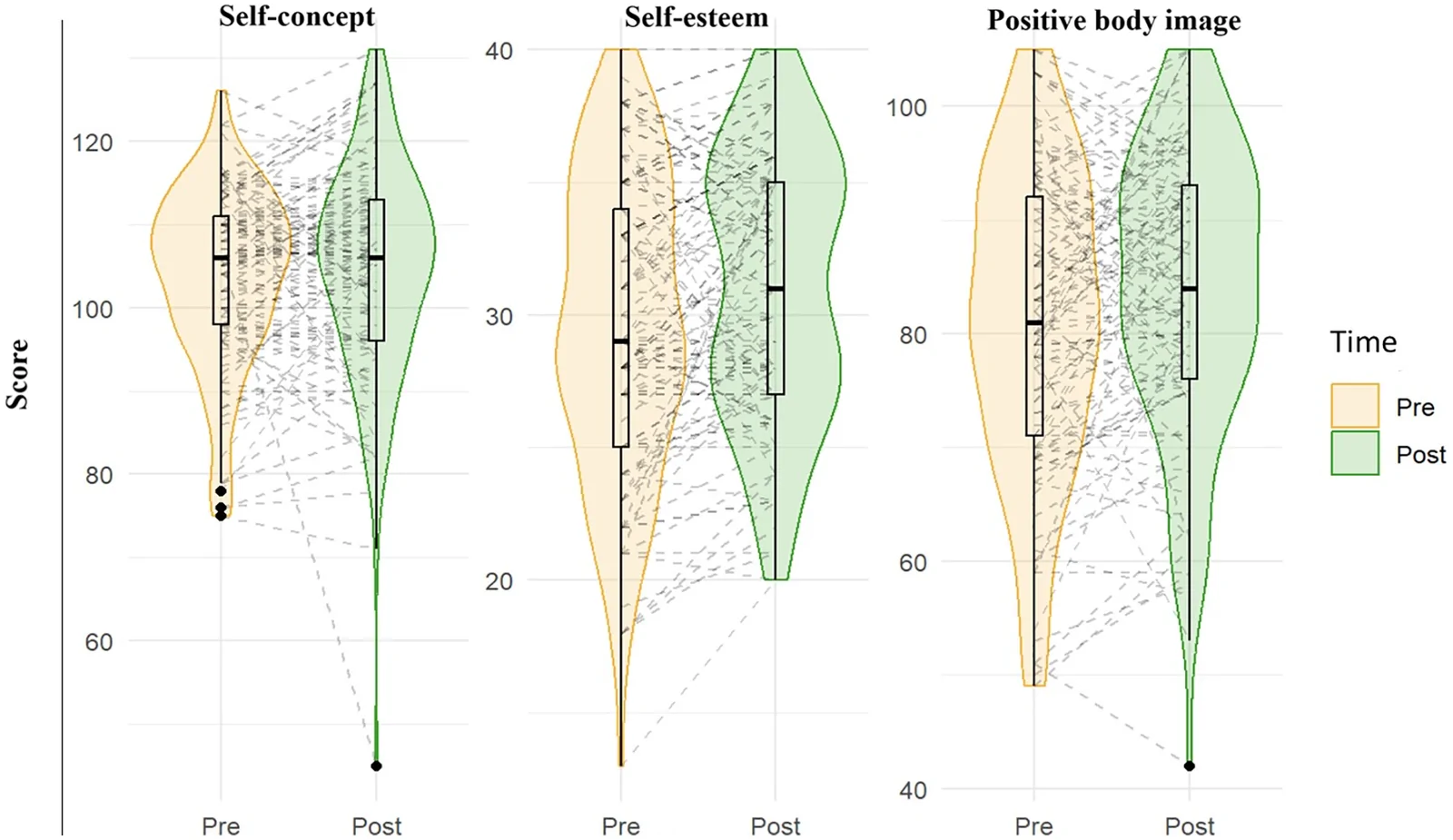
Comparison of main variables.

Self-esteem also increased, from 29.00 [25.00, 34.00] at preintervention to 31.00 [27.00, 35.00] at postintervention. This difference was statistically significant (V = 3543.5, p < .001) and was associated with a moderate effect size (r_rb = .432, 95% CI [.241,  .590]).

For self-concept, the median score was 106.00 [98.00, 111.00] at preintervention and 106.00 [96.00, 113.00] at postintervention. The paired rank-based analysis yielded a small, borderline pre–post difference (V = 3396.5, p = .050, r_rb = .221, 95% CI [.008,  .414]).

Individual pre–post change scores did not differ significantly between girls and boys for positive body image (U = 998.0, p = .985), self-esteem (U = 1088.0, p = .511), or self-concept (U = 1100.5, p = .568). Thus, there was no evidence that the magnitude of pre–post change differed by sex for any of the three main outcomes.

### Comparison of PBIAS subscales

Exploratory analyses of the PBIAS subscales are presented in Table 5 and Fig 3. A statistically significant pre–post difference was observed only for resilience to negative appearance feedback, for which the median increased from 4.92 [3.87, 5.70] to 5.47 [4.25, 6.26] (V = 3784.0, p < .001, r_rb = .441, 95% CI [.250,  .598]).

**Table 5:** Pre–post changes in Positive Body Image in Adolescents Scale subscales and differences in change by sex.

PBIAS subscale	*n*	Pre, Md [Q1, Q3]	Post, Md [Q1, Q3]	Change, Md [Q1, Q3]	*V*	*p*	*r*rb [95% CI]	Girls change, Md [Q1, Q3]	Boys change, Md [Q1, Q3]	*U*	*p*	*r*rb [95% CI]
Own-body appreciation	107	5.65 [4.67, 6.50]	5.82 [4.77, 6.25]	0.02 [−0.33, 0.42]	2787.0	0.593	0.061 [−0.156, 0.272]	0.01 [−0.34, 0.35]	0.05 [−0.32, 0.50]	1036.0	0.508	−0.084 [−0.320, 0.162]
Others’ body appreciation	105	7.00 [6.00, 7.00]	7.00 [6.03, 7.00]	0.00 [0.00, 0.35]	994.0	0.413	0.123 [−0.096, 0.331]	0.00 [0.00, 0.24]	0.31 [−0.67, 1.02]	870.5	0.163	−0.173 [−0.404, 0.079]
Resilience to media body ideals	109	5.00 [4.00, 6.00]	5.51 [4.00, 6.00]	0.02 [−0.51, 1.00]	2560.5	0.217	0.147 [−0.068, .349]	0.02 [−0.51, 1.00]	0.00 [−1.02, 0.89]	1072.0	0.445	0.095 [−0.147, 0.327]
Resilience to negative appearance feedback	108	4.92 [3.87, 5.70]	5.47 [4.25, 6.26]	0.46 [−0.22, 1.15]	3784.0	< 0.001	0.441 [0.250, 0.598]	0.52 [−0.19, 1.13]	0.39 [−0.44, 1.66]	1117.5	0.849	0.024 [−0.219, 0.265]

Values are presented as median [first quartile, third quartile]. Change was calculated as postintervention minus preintervention for each participant. Overall pre–post changes were assessed using two-sided Wilcoxon signed-rank tests. Differences in individual change scores between girls and boys were assessed using two-sided Mann–Whitney U tests. rrb = rank-biserial correlation; CI = confidence interval; PBIAS = Positive Body Image in Adolescents Scale.

**Fig 3:**
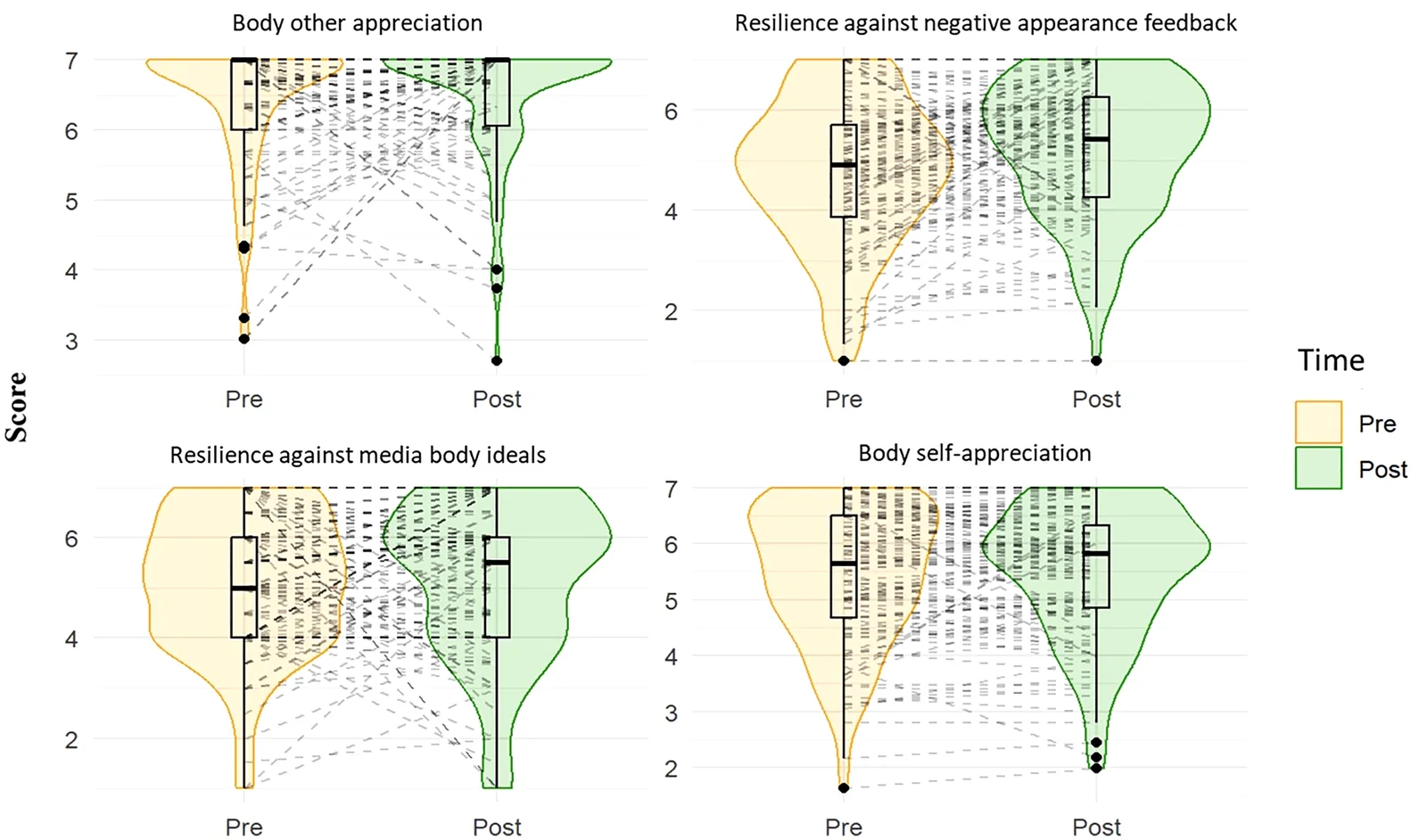
Comparison of PBIAS subscales.

No statistically significant pre–post differences were observed for own-body appreciation (V = 2787.0, p = .593), others’ body appreciation (V = 994.0, p = .413), or resilience to media body ideals (V = 2560.5, p = .217).

There was also no evidence that individual pre–post changes differed between girls and boys for own-body appreciation (U = 1036.0, p = .508), others’ body appreciation (U = 870.5, p = .163), resilience to media body ideals (U = 1072.0, p = .445), or resilience to negative appearance feedback (U = 1117.5, p = .849).

## Discussion

This study aimed to assess the changes observed following the implementation of a community co-created intervention programme on screen use, self-esteem, self-concept, and positive body image among adolescents from a rural area in Catalonia. Community-based interventions have proven to be a key strategy in promoting adolescent health, as they allow for a more contextualised and participatory approach. The principal findings were modest improvements in overall positive body image and self-esteem. The rank-based effect size for positive body image was small-to-moderate, whereas the improvement in self-esteem was of moderate magnitude. Exploratory analyses further suggested that the observed improvement in positive body image was concentrated primarily in resilience to negative appearance feedback.

Self-concept showed a small and borderline pre–post difference. Although the rank-biserial confidence interval excluded zero, the p value was at the conventional significance threshold and several outcomes were examined. This result should therefore be interpreted cautiously and does not provide strong evidence of a meaningful change in self-concept.

No evidence was found that pre–post changes in positive body image, self-esteem, self-concept, or any of the PBIAS subscales differed between girls and boys. Accordingly, the present analyses do not support a differential response to the programme according to sex. This finding concerns differences in change over time and should be distinguished from cross-sectional sex differences in outcome levels.

The preventive approach of this study made it difficult to directly compare its results with those of other studies. Most community interventions have focused on the pathology of adolescents’ physical and mental health. These interventions, primarily carried out within the school setting, addressed a wide range of objectives, and their effectiveness has been limited due to the methodological heterogeneity applied and the cultural diversity of participants [42]. Therefore, the need to design interventions that not only seek to reduce risks but also actively promote protective factors such as the development of high self-esteem, a positive self-concept, positive body image, and greater awareness of the impact of screen use on mental health has been emphasised.

Previous studies have proposed the active participation of adolescents in the development of interventions [43,44]; however, this study aimed to include all relevant stakeholders, such as parents and educators, who influence adolescent development. To our knowledge, this is the first study to assess the changes in self-esteem, self-concept, and positive body image among adolescents, following the implementation of an intervention program created in collaboration with the rural community, specifically designed to promote healthy screen use among adolescents.

Our results show that after implementing a co-created community intervention program focused on screen use, self-esteem and positive body image improve, with the improvement in positive body image appearing to be driven primarily by resilience to negative appearance feedback, suggesting that this innovative approach may have a positive impact on adolescents’ well-being and their environment.

Among the PBIAS dimensions, the clearest change was observed for resilience to negative appearance feedback. This finding may be particularly relevant in adolescence, when appearance-related comments, social comparison, and digital interactions can contribute to body-image concerns. However, because the subscale analyses were exploratory and no adjustment for multiple testing was applied, the finding should be interpreted as hypothesis-generating rather than confirmatory.

Regarding self-concept, baseline scores were higher in girls than in boys, and both groups showed a tendency towards improvement after the intervention. This finding is interesting and contrasts with previous studies, which suggested that self-concept is closely related to both self-esteem and positive body image.

Several authors have emphasised that body image is one of the most important components of an individual's self-concept, and that the perception of one's own body and the associated feelings strongly influence not only self-concept but also self-esteem [20,45]. However, the results of this study suggest that other factors, apart from body image and self-esteem, may be influencing the construction of self-concept in adolescents.

Self-concept is a multidimensional construct dependent on perceived positive or negative personal experiences, as well as various biological, psychological, and sociocultural factors. In adolescence, this process is strongly influenced by the family and school environment, particularly by the expectations and judgments of significant others (parents, siblings, and peers) and the interactions between them [46]. Gallardo [47] suggests that the quality of attachment established with parental figures during early childhood determines the degree of positivity or negativity of self-concept. Moreover, in the school context, the role of the teacher and the level of expectations placed on the student are also determinants in the construction of a favourable or unfavourable self-concept [47]. This suggests that self-concept may be influenced by a broader set of experiences and factors that are not always reflected in interventions focused exclusively on positive body image or self-esteem. Therefore, interventions aimed at improving self-concept should address not only these aspects but also the role of family and school expectations, as well as the quality of relationships and attachment. In this regard, it is essential to design interventions that foster positive relationships, promote secure attachment, and strengthen family bonds, building trust in social and school relationships. In the latter, interactions with teachers and peers play a key role, as teachers not only impart knowledge but also influence the perception that adolescents have of their own abilities through the expectations they place on them and the recognition or reinforcement they provide [46,48,49].

Regarding sex differences, the results of this study did not reveal sex-specific effects regarding the intervention; however, cross-sectional differences in self-esteem and positive body image scores were observed at baseline. Boys showed higher scores in both variables, which aligns with existing literature. There is evidence showing that girls experience greater dissatisfaction with body image [50, 51] due to social pressure and beauty ideals promoted by the media and social networks [52,53]. This finding reinforces the idea that girls, compared to boys, may be more influenced by these standards, leading to greater dissatisfaction with their body image and lower self-esteem.

These sex differences may be explained by the different appearance-related pressures experienced during adolescence. Compared with boys, girls are more frequently exposed to sociocultural norms that emphasise thinness, physical attractiveness, and appearance as central components of personal and social value. These pressures are reinforced by family, peers, traditional media, and particularly social media, where appearance-focused content and social comparison are especially prevalent among adolescent girls [54,55]. According to the Tripartite Influence Model [56] and Objectification Theory [57], these influences promote the internalisation of appearance ideals, body surveillance, and appearance-based social comparison, increasing vulnerability to body dissatisfaction and lower self-esteem. Our findings therefore support previous evidence indicating that adolescent girls constitute a particularly vulnerable group regarding body image concerns [54,55], highlighting the importance of incorporating a sex-sensitive perspective into interventions promoting healthy screen use and positive body image.

Another relevant finding was the observed differences in the scores of the subscales of the PBIAS, such as self-appreciation, appreciation of others, and resilience towards media. Boys, compared to girls, showed greater self-appreciation and less appreciation for others, suggesting that they are less vulnerable to internalising the beauty standards and ideals promoted by the media, in line with existing literature [53,58]. This could be a result of lower social pressure on males to comply with strict aesthetic stereotypes, which may lead to greater self-acceptance and less social comparison. At the same time, this difference may indicate that boys are less exposed to the externalisation of their personal value based on appearance, which could result in a more stable self-esteem. These findings are consistent with previous studies suggesting that boys tend to base self-worth on a broader range of domains, such as athletic competence or physical functionality, whereas girls are more likely to evaluate themselves according to physical appearance and perceived attractiveness. Consequently, girls may be more susceptible to appearance-based social comparison and external validation, particularly in image-centred online environments [59].

Regarding resilience to the media, boys and girls did not differ in this dimension. Nevertheless, this study suggests that the intervention may have helped adolescents develop a greater critical ability to handle external influences related to media messages, whereas the improvement observed over time was concentrated in resilience to negative appearance feedback. This is particularly relevant, as resilience to criticism about appearance is crucial for protecting adolescents’ mental health and self-esteem in a digital environment saturated with comparisons and judgments [60].

Similarly, no sex differences were found in resilience to negative appearance feedback, which refers to how the adolescent responds and adapts to comments and reactions they receive from others about their body. This capacity enables the maintenance of a positive body image despite direct social interactions, and it is key to preserving self-esteem and emotional well-being. Resilience to feedback can be understood as a specific manifestation of psychological resilience, which is determined by the interactions between internal and external factors [61]. Internal determinants include psychological and biological factors, while external ones include the quality and nature of the environment, as well as relationships inside and outside the family [61]. In this regard, Çakmak and Bolluk [62] found that high family expectations and parental attitudes of support, care, and warmth have positive effects on psychological resilience. Thus, parental attitudes of support, authority, democracy, and care generate or increase psychological resilience, while negligent and hostile parental attitudes negatively affect adolescents’ psychological resilience [62]. This argument reinforces the proposal to include parents in interventions, as was done in this study, and to work on specific issues such as attachment, which has proven its relationship with both self-concept and resilience. Furthermore, interventions aimed at adolescents should be adapted to the specific experiences and needs of each sex. Although the intervention produced comparable changes in girls and boys, girls and boys internalise and respond to social expectations differently, which may explain the baseline differences observed in this study and supports tailoring intervention content to these distinct experiences.

Thus, community co-created interventions can have a positive effect and, at the same time, offer a comprehensive approach that considers the family, school, and social dimensions in the execution of interventions for adolescent development. This approach proves promising in improving adolescents’ psychosocial well-being, as it addresses the various influences shaping their daily experience and reality, especially in rural contexts. Rural environments differ from urban ones due to health peculiarities [63] and a lack of resources [64], where social pressures may intensify the negative effects on adolescents’ self-esteem, self-concept, and positive body image. The participatory approach adopted has proven effective in promoting positive change in self-esteem and positive body image by adapting strategies to the specific sociocultural characteristics of the community [30,31].

Future studies should continue investigating how co-created interventions can be optimised to improve self-esteem, self-concept, and positive body image in adolescents, particularly among girls, who appear to be more exposed to social pressures regarding appearance. Furthermore, it would be relevant to analyse the long-term impact of these strategies and their potential to generate sustainable changes in adolescents’ attitudes and behaviours, particularly in areas such as screen use and social media.

### Strength and limitations

This study represents the first community-designed and implemented intervention aimed at improving self-esteem, self-concept, and positive body image in adolescents. A major strength of this study is the use of a participatory approach, which actively involved key community stakeholders throughout the development process, allowing the intervention to be adapted to local needs, resources, and priorities. Additionally, the intervention was developed and implemented under real-world community conditions, providing valuable insights into the feasibility of delivering a health promotion programme within routine practice in a rural setting. Furthermore, the combination of scientific evidence, community perspectives, and clinical expertise during the intervention development process represents a strength, as it enabled the design of a contextually appropriate programme that may inform the development of similar community-based initiatives in comparable settings.

However, several limitations must be acknowledged. The lack of direct communication between the school nurse and parents may have contributed to the low participation rate. Furthermore, the absence of a control group prevents us from attributing observed changes solely to the intervention, as other uncontrolled factors (e.g., developmental maduration, school-related influences, seasonal effects, or concurrent psychoeducational activities) may have influenced the results. Additionally, the intervention comprised multiple components (e.g., community meetings, workshops, theatre performance, family sessions, and school-based activities), which reflects the complexity of community-based interventions but limits the ability to determine the contribution of each individual component or identify specific mechanisms underlying the observed changes.

Moreover, although the intervention targeted the broader community, data collection focused exclusively on adolescents, limiting the assessment of its impact on parents and educators. In addition, the relatively low participation rate (109 out of 398 eligible students) should be considered when interpreting the findings, as it may introduce potential selection bias due to the voluntary nature of participation and parental consent procedures. Families with greater interest in mental health or digital well-being issues may have been more likely to participate, meaning that participating adolescents may differ from non-participants in ways that could influence the observed outcomes.

Given that the study was conducted in a single rural community in Catalonia, the transferability and generalizability of the findings to other cultural, socioeconomic, or educational contexts should be interpreted with caution.

Future research should consider expanding the study to other municipalities using socioeconomic indices like the MEDEA index, which would allow for comparisons across regions with differing socio-economic contexts, thereby enhancing the generalizability of the findings. Additionally, future research should explore long-term effects and adaptation to diverse populations, considering factors such as culture, gender, and rural or urban settings. Future studies should also incorporate objective measures of screen use (e.g., digital tracking or device-based logs) to better capture behavioral changes and strengthen inferences regarding associations between the intervention and digital behaviors when feasible in real-world community settings.

## Conclusions

The results of this study suggest that community co-created interventions may be a promising strategy for promoting self-esteem and positive body image among adolescents, particularly in rural settings where mental health resources may be more limited. This participatory approach not only offers a more inclusive and sustainable intervention model but may also contribute to strengthening the integration and collaboration among the various systems influencing adolescents’ psychosocial well-being, such as family, school, and community. It is hoped that the findings will encourage health professionals, educators, and policymakers to design strategies and intervention programmes that promote responsible digital habits among young people, as well as high self-esteem and positive body image, taking into account the specific needs of the adolescent population in Catalonia.

## Abbreviations

CSE: Compulsory Secondary Education
DCN: Data Collection Notebook
PBIAS: Positive Body Image in Adolescents Scale
